# Host and Viral Modulation of RIG-I-Mediated Antiviral Immunity

**DOI:** 10.3389/fimmu.2016.00662

**Published:** 2017-01-03

**Authors:** Yiliu Liu, David Olagnier, Rongtuan Lin

**Affiliations:** ^1^Jewish General Hospital, Lady Davis Institute, McGill University, Montreal, QC, Canada; ^2^Division of Experimental Medicine, McGill University, Montreal, QC, Canada; ^3^Department of Microbiology and Immunology, McGill University, Montreal, QC, Canada

**Keywords:** innate immunity, antiviral, infection, RIG-I, type I IFNs, virus–host interaction

## Abstract

Innate immunity is the first line of defense against invading pathogens. Rapid and efficient detection of pathogen-associated molecular patterns *via* pattern-recognition receptors is essential for the host to mount defensive and protective responses. Retinoic acid-inducible gene-I (RIG-I) is critical in triggering antiviral and inflammatory responses for the control of viral replication in response to cytoplasmic virus-specific RNA structures. Upon viral RNA recognition, RIG-I recruits the mitochondrial adaptor protein mitochondrial antiviral signaling protein, which leads to a signaling cascade that coordinates the induction of type I interferons (IFNs), as well as a large variety of antiviral interferon-stimulated genes. The RIG-I activation is tightly regulated *via* various posttranslational modifications for the prevention of aberrant innate immune signaling. By contrast, viruses have evolved mechanisms of evasion, such as sequestrating viral structures from RIG-I detections and targeting receptor or signaling molecules for degradation. These virus–host interactions have broadened our understanding of viral pathogenesis and provided insights into the function of the RIG-I pathway. In this review, we summarize the recent advances regarding RIG-I pathogen recognition and signaling transduction, cell-intrinsic control of RIG-I activation, and the viral antagonism of RIG-I signaling.

## Introduction

Eukaryotic organisms rely on the host innate immune system to defend against viruses or other pathogenic microbes in early phases of infection. The innate antiviral immune response starts with the detection of evolutionarily conserved structures, termed pathogen-associated molecular patterns (PAMPs), by a set of germline-encoded pattern-recognition receptors (PRRs). With respect to their cellular localization, ligand specificity, and functions, PRRs are categorized into distinct families including the toll-like receptors, nucleotide-binding oligomerization domain-like receptors, C-type lectin receptors, retinoic acid-inducible gene-I (RIG-I)-like receptors (RLRs) (1–5), as well as cytosolic viral DNA sensors such as cyclic GMP-AMP synthase (6, 7). Following the detection of specific viral PAMPs, PRRs trigger the activation of intracellular signaling cascades, ultimately leading to the production of type I interferons (IFNs), as well as pro-inflammatory cytokines. Secreted IFNs are crucial for the induction of numerous interferon-stimulated genes (ISGs); the products of which are major forces in controlling and restricting viral infections, thereby establishing a cellular antiviral state as well as helping to shape the adaptive immune response (8). Recent studies showed that viruses have evolved complex strategies to affect multiple stages of the host antiviral defense, from inhibiting the viral detection to manipulating components of the signaling pathways (9, 10). To ensure successful antiviral defenses and to avoid aberrant or dysregulation of host immune signaling, antiviral pathways need to be tightly regulated at each level. In this review, we will summarize the cell-intrinsic regulation of RIG-I receptor activity, as well as the viral strategies to subvert the RIG-I signaling machinery.

## RIG-I Structure and Ligand Interactions

**Figure 1:**
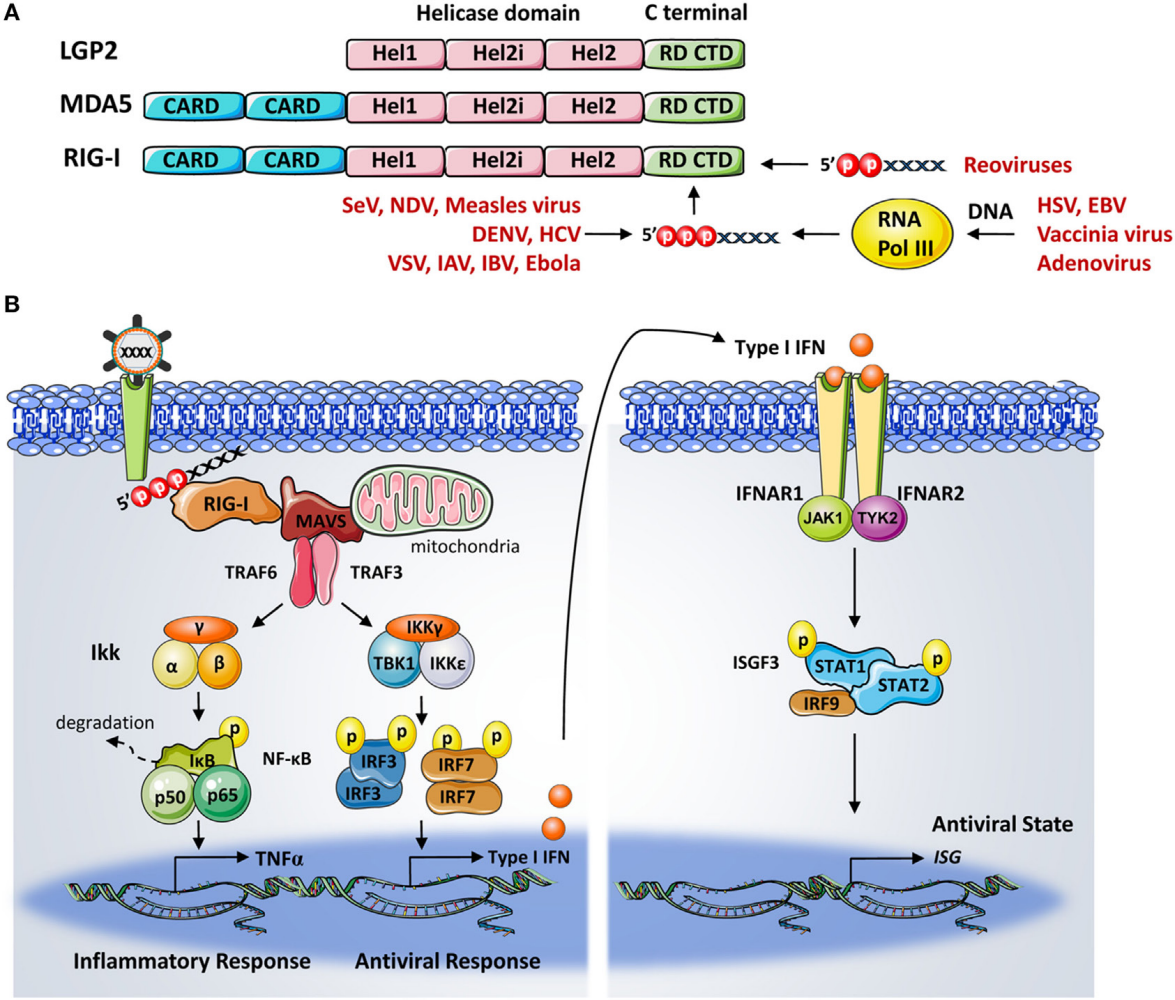
**(A)** Domain structure of retinoic acid-inducible gene-I (RIG-I). RIG-I belongs to the DExD/H-box family of helicases and is comprised of helicase domains 1 (Hel1) and 2 (Hel2) with a Hel2i insertion, N-terminal caspase activation and recruitment domains, and a C-terminal domain (CTD) or repressor domain. RIG-I CTD is responsible for recognizing a plethora of RNA viruses with short 5′ triphosphate (5′ppp) RNA and 5′-diphosphate-bearing RNA structures. RIG-I also detects 5′ppp RNA species synthesized through the transcription of viral DNA by RNA polymerase III. **(B)** The cytoplasmic pattern-recognition receptor RIG-I is essential for recognizing RNA viruses with a 5′ppp signature. Upon viral RNA recognition, RIG-I recruits the adaptor protein mitochondrial antiviral signaling protein to activate the TBK1–IKKϵ complex and IKKα–IKKβ complex, which are responsible for the activation of transcription factors interferon regulator factor (IRF) 3, IRF7, and nuclear factor-κB. These transcription factors then translocate to the nucleus and coordinate the induction of type I interferons (IFNs). This is followed by the binding of the IFNs α and β to their cognate receptor, which will lead to the transcriptional activation of interferon-stimulated genes (ISGs) by the JAK/STAT signaling pathway. The products of ISGs are key factors in limiting pathogen spreading.

The three members of the RLR family: RIG-I, MDA5 (melanoma differentiation factor 5), and LGP2 (laboratory of genetics and physiology 2) are expressed in most cell and tissue types. They function as cytoplasmic sensors for the recognition of a variety of RNA viruses and subsequent activation of downstream signaling to drive type I IFN production and antiviral gene expressions. These three RLR proteins are RNA-dependent ATPases belonging to the DExD/H-box family of helicases (11). Structurally, RLRs have a similar central helicase core that is comprised of two helicase domains, Hel1 and Hel2 with an insertion termed Hel2i. In addition, they all have a C-terminal domain (CTD). However, only RIG-I and MDA5 contain two N-terminal caspase activation and recruitment domains (CARDs) (3) (Figure 1A). Among these three, RIG-I is the founding member and hence the most intensively studied member of this family. Each domain of RIG-I plays unique roles during RIG-I autorepression and activation. In brief, the CTD and helicase domain are involved in RNA ligand binding and ATP hydrolysis-involved conformational changes (12–14), whereas the RIG-I CARDs facilitate interaction with other downstream CARD containing molecules (15).

Retinoic acid-inducible gene-I has been shown to be involved in the recognition of a variety of RNA viruses in the cytoplasm, such as the Sendai virus, influenza A and B viruses (IAV, IBV), vesicular stomatitis virus, measles virus (MV), Newcastle disease virus, Ebola virus (EBOV), dengue virus (DENV), and hepatitis C virus (HCV) (16–19). The short double-stranded (ds) RNA with a triphosphate (ppp) motif at the 5′-end, as found in these viral genomes, were shown to be a key signature recognized by RIG-I (20, 21). The 5′ppp dsRNA of viral nucleocapsids has also been characterized as stimulating RIG-I (22). 5′-Diphosphate-bearing RNA (5′ppRNA), either naturally contained in viruses, produced by *in vitro* transcription, or *via* chemical synthesis, were all shown to bind to RIG-I and were sufficient to activate RIG-I (20, 23). Physiologically, the control of *in vitro* and *in vivo* infections of reoviruses, which bear the 5′ppRNA genome, relies on RIG-I functionality (24). It is worth noting that the *in vitro-*synthesized 5′pppRNA sequences also trigger RIG-I activation (25). These agonists have demonstrated their therapeutic potential as broad-spectrum antiviral agents and could be optimized as vaccine adjuvant candidates (26–30). Furthermore, the recognition of several DNA viruses, including herpes simplex virus type 1 (HSV-1), Epstein–Barr virus (EBV), vaccinia virus (VACV), and adenovirus, *via* the RNA polymerase III were found to be RIG-I-dependent (31, 32). Interestingly, the RIG-I-mediated upregulation of STING is required for protection against the HSV-1 by the RIG-I agonist, offering new evidence of the overlapping between RIG-I signaling and the host response to DNA viral infection (33). Notably, viral RNA triggered RIG-I signaling also mediates the inflammatory response *via* distinct pathways. The first involves the formation of the RIG-I inflammasome through interactions between RIG-I, ASC, and caspase-1 and the stimulation of IL-1β release. The second involves the adaptor proteins CARD9, Bcl-10, mitochondrial antiviral signaling protein (MAVS), and the activation of nuclear factor-κB (NF-κB) (34, 35). Upon RNA ligand binding, RIG-I undergoes a series of conformational changes and posttranslational modifications (PTMs) to achieve full activation (further detail below).

## RIG-I Signaling Transduction

Activated RIG-I recruits its downstream adaptor molecule MAVS (also known as IPS-1, CARDIF, and VISA) through CARD–CARD-mediated interactions (36, 37). The oligomeric RIG-I CARD assembly and the polymeric formation of MAVS, together serve as a signaling platform for protein complexes that mediate the bifurcation of signaling into two branches. One branch recruits tumor necrosis factor receptor-associated factors (TRAF)-2/6 and the receptor-interacting protein 1 to subsequently activate the IKK complex, resulting in NF-κB activation (38). The other branch signals through TRAF3 and activates the TANK/IKKγ/IKKϵ/TBK1 complex, leading to the phosphorylation and dimerization of interferon regulator factors (IRF)-3 and -7 (39, 40). Activated IRF3/7 and NF-κB then translocate to the nucleus, together with ATF2, c-Jun, and the transcription coactivator CREB-binding protein/p300, to coordinate the IFN and pro-inflammatory gene expressions (41). Once secreted, IFNs bind to specific cell surface receptors and activate the JAK–STAT pathway. The activated transcription factors STAT1, STAT2, and IRF9 form the interferon-stimulated gene factors (ISGF3) complex. ISGF3 then translocates to the nucleus and coordinates the transcription of hundreds of ISGs including RIG-I, thus generating an amplifying loop leading to the accumulation of RIG-I during several types of infections (8) (Figure 1B).

## Mechanisms of RIG-I Activation

### RIG-I Autorepression

Structural and biochemical studies have demonstrated that the activation of RIG-I is a multi-step process and is primarily regulated by conformational changes and PTMs. When initially identified as a dsRNA sensor, it was hypothesized that RIG-I was under negative regulation in physiological conditions. The over expression of the CARD domain of RIG-I alone demonstrated superior signaling activity than full length RIG-I in absence of viral PAMPs (2). Studies by Saito et al. showed that the deletion of CARD was dominant-negative for RIG-I signaling. By contrast, the deletion of repressor domain (RD) resulted in constitutive signaling, whereas RD expression alone ablated RIG-I signaling actions. Together, these findings provided the model of RIG-I autoregulation in which the RD is predicted to mask CARDs for signaling transduction in uninfected cells (42). The crystal structural analysis further delineated the models of autorepressed and ligand activated states of RIG-I, respectively. In a ligand-free state, CARDs and Hel2i interactions hinder dsRNA binding and inactivate RIG-I (14). The binding of 5′ppp dsRNA to RD leads to a conformational switch of RIG-I, which releases the autorepressed CARDs and exposes the helicase domain for ATP binding (14, 43). ATP hydrolysis is essential for RIG-I signaling. It enables RIG-I to translocate along the dsRNA, and further promotes the oligomerization of RIG-I CARDs. These processes assemble RIG-I into a filamentous architecture which facilitates the CARD–CARD interactions with the mitochondrial MAVS, leading to the subsequent signaling transduction for IFN production (44, 45). Importantly, RIG-I ATPase activity also plays a role in distinguishing self-RNA from non-self-RNA (46). It was reported that RIG-I ATP hydrolysis increases the binding affinity of RIG-I and dsRNA ligands; whereas the RIG-I mutants deficient in ATP hydrolysis promotes the interaction of RIG-I and self-dsRNA and results in unintentional immune signaling (47).

### Posttranslational Control of RIG-I

#### Ubiquitination

**Figure 2:**
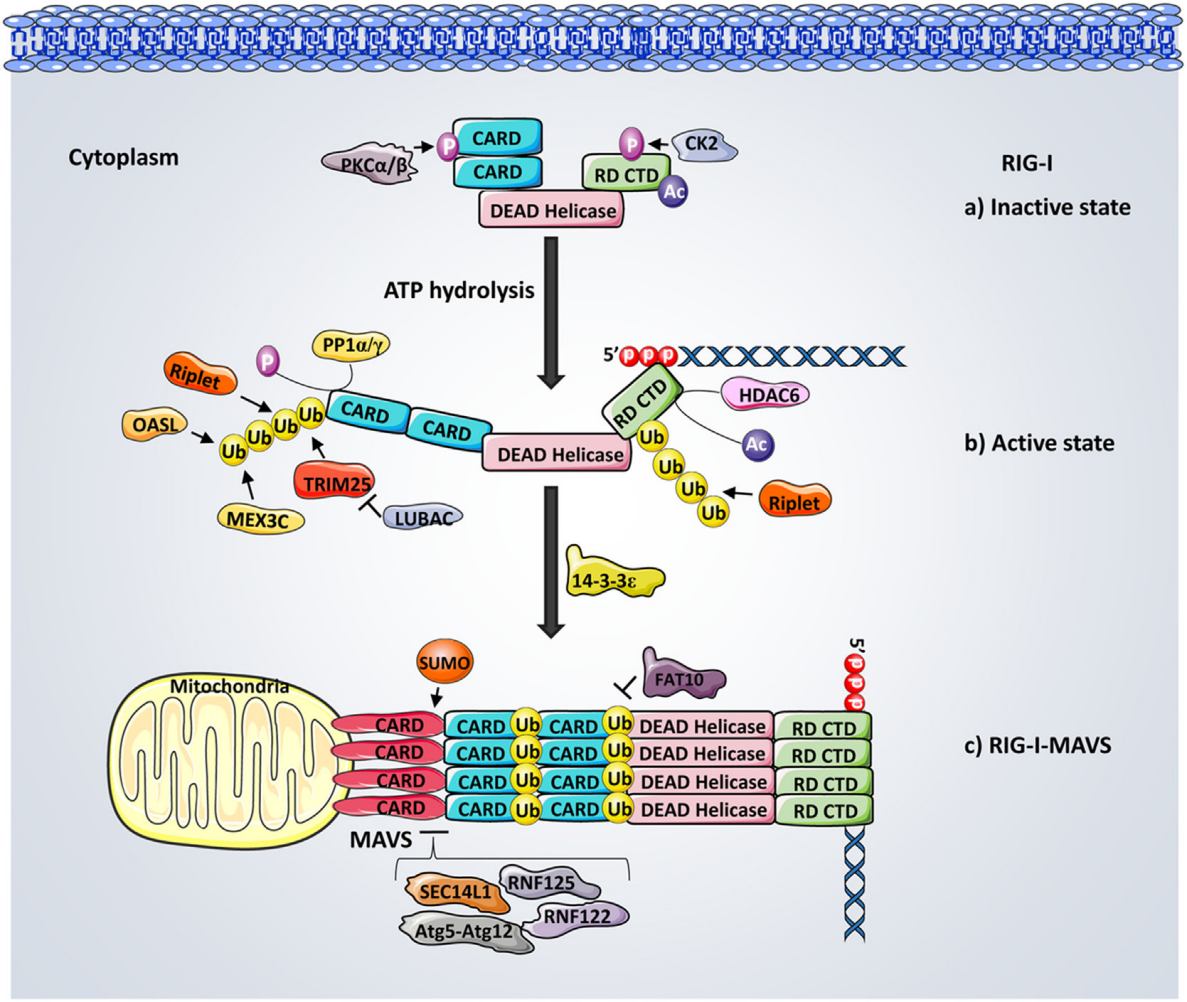
**Regulation of retinoic acid-inducible gene-I (RIG-I) activation**. (a) In resting cells, RIG-I is kept inactivated through the phosphorylation of caspase activation and recruitment domains (CARDs) and C-terminal domain (CTD) mediated by casein kinase II and protein kinase C-α/β, respectively. (b) Following the binding of 5′ triphosphate (5′ppp) RNA and ATP hydrolysis, RIG-I is dephosphorylated by phosphoprotein phosphatase 1-α/γ and results in a conformational change that opens CARDs. HDAC6-mediated deacetylation of RIG-I CTD is critical for RIG-I and 5′pppRNA binding. The Lys63-linked ubiquitination of RIG-I mediated by TRIM25, Riplet, oligoadenylate synthetases-like protein, and MEX3C at both CARDs and CTD further activate RIG-I and facilitate its tetramerization. (c) Interactions between RIG-I–TRIM25 complex and 14-3-3ϵ promote RIG-I translocation to mitochondrial mitochondrial antiviral signaling protein (MAVS) for downstream signaling, leading to interferon production. Interactions between TRIM25, RIG-I, and MAVS are further negatively regulated by the Lys48-linked ubiquitination, which is meditated by LUBAC, RNF125, and RNF122. SEC14L1 and Atg5–Atg12 both inhibit the signaling by interrupting RIG-I–MAVS interactions, whereas SUMOylation promotes RIG-I–MAVS binding.

One of the first PTMs of RIG-I following the initial ligand recognition is performed by the robust ubiquitination machinery (Figure 2). Mass spectrometry analysis revealed that TRIM25, a member of the tripartite motif (TRIM) protein family possessing E3 ligase activity, induces the covalent Lys63-linked ubiquitination of RIG-I. Mechanistically, the C-terminal SPRY domain of TRIM25 interacts with CARD1 and facilitates the ubiquitination of CARD2 at K172 (48). The RIG-I–TRIM25 ubiquitination complex, associates with the adaptor protein 14-3-3ϵ and translocates to mitochondria for MAVS binding (49). Mutation of K172 disrupts the interaction between RIG-I and MAVS thus abrogating downstream signaling and IFNs production (50). Furthermore, a RIG-I splice variant which lacks the TRIM25 interaction domain acts as a feedback inhibitor of RIG-I signaling transduction upon viral infections (48). In addition, Riplet (RING-finger protein leading to RIG-I activation, also named RNF135 or REUL), another E3 ubiquitin ligase, also promotes RIG-I ubiquitination. Multiple sites within the CARDs, as well as within the CTD of RIG-I, were identified as the crucial ubiquitin anchoring residues (51–53). Among which, K63-linked polyubiquitination (pUb) at Lys788, is demonstrated as being critical for RIG-I activation. However, unlike TRIM25-induced ubiquitination, Riplet induced RIG-I pUb is dispensable for RIG-I-RNA binding but is essential for releasing CARD from its autorepressed state. This enhances TRIM25 functionality as well as promoting the oligomerization of RIG-I and the activation of MAVS (54). MEX3C (Mex-3 RNA binding family member C), another recently identified E3 ligase, also mediates Lys63-Ub at K99 and K169 of CARD, playing a critical role in RIG-I activation (55). In addition, the oligoadenylate synthetases-like (OASL) protein, although not an E3 ubiquitin ligase itself, contains a dsRNA-binding groove and enhances RIG-I activation by mimicking the K63-linked pUb through its ubiquitin-like (UBL) domain (56, 57). Non-covalent binding of K63-ubiquitin chains to CARDs also potently activates RIG-I (58). Recent structural analysis suggests that covalent and non-covalent binding of ubiquitin synergistically stabilize RIG-I tetramerization and enhance polymerization of MAVS CARDs (59).

On the other hand, several deubiquitinating enzymes (DUBs) were identified to remove K63-linked pUb chains from RIG-I, thus dampening RIG-I signaling. The tumor suppressor protein cylindromatosis (CYLD) removes K63-linked pUb chains from RIG-I as well as TBK1 and IKKϵ to inhibit the IRF3 response, serving as a pathway negative regulator (60). Syndecan-4, a newly identified negative regulator of RIG-I, functions through attracting CYLD to RIG-I complex, thus potentiating the K63-mediated deubiquitination of RIG-I (61). In addition, the ubiquitin-specific protease (USP) family members, such as USP3 and USP21, were also identified as inhibitors of RIG-I activation by deubiqutinating RIG-I (62, 63).

In contrast to K63-linked ubiquitination, which promotes protein activation, K48-linked ubiquitination triggers proteasomal degradation of its target. For instance, the RING-finger protein 125 (RNF125), together with the ubiquitin E2 ligase UbcH5, conjugate K48-linked ubiquitin to RIG-I and MAVS, targeting them for proteasomal degradation and thereby inhibiting downstream signaling (64). Similarly, RNF122 was recently demonstrated to mediate the proteasomal degradation of RIG-I by delivering the K48-linked ubiquitin to RIG-I CARDs (65). The linear ubiquitin assembly complex (LUBAC) has been shown to promote K48 pUb of TRIM25, leading to its degradation (66). Conversely, the deubiquitinase USP15 antagonizes LUBAC by removing K48-linked ubiquitin from TRIM25, leading to its stabilization and thereby promoting RIG-I-mediated antiviral signaling (67).

#### Phosphorylation

In parallel with ubiquitination, phosphorylation has emerged in the past several years as a critical regulator of the RIG-I signaling transduction (Figure 2). Protein purification and mass spectrometry analysis identified that phosphorylation of Thr170 in the CARDs antagonizes RIG-I signaling by inhibiting TRIM25-mediated Lys172 ubiquitination and MAVS binding (68). Ser8 phosphorylation of CARDs also serves as a negative regulator of RIG-I (69). In addition, the CTD of RIG-I is constitutively phosphorylated at Thr770 and Ser854/855 by casein kinase II to promote intermolecular interactions between CTD and CARDs, thereby maintaining RIG-I at an autorepressive state to prevent premature downstream signaling (70). A recent mass spectrometry analysis revealed that IKK phosphorylates RIG-I at Ser855, thereby providing a negative feedback regulation of RIG-I (71). Furthermore, conventional protein kinase C-α (PKC-α) and PKC-β have also been shown to phosphorylate CARDs, thus suppressing RIG-I–TRIM interaction and subsequent antiviral responses (72). In fact, RIG-I signaling activity is controlled by a dynamic balance between phosphorylation and dephosphorylation. Dephosphorylation of RIG-I occurs rapidly with the presence of viral RNA. A functional siRNA screen identified phosphoprotein phosphatase 1-α (PP1α) and PP1γ as essential phosphatases responsible for CARDs dephosphorylation at Ser8 and Thr170, leading to RIG-I signal activation and viral inhibition (73).

#### Acetylation

In addition to the ubiquitination and phosphorylation described above, acetylation modulation has recently started to gain more acknowledgment for controlling RIG-I activity (Figure 2). Mass spectrometry has identified the acetylation of two lysine residues (K858 and K909) in the CTD of RIG-I at its inactivate state and are deacetylated during viral infection (74). The mutation of these two sites restricts RIG-I from undergoing the virus-induced interaction with MAVS. K858 and K909 acetylation of RIG-I has also been shown to control the PAMP RNA-induced RIG-I oligomerization (75). The cytoplasmic deacetylase HDAC6-mediated removal of K909 acetylation has been shown as critical for RIG-I binding to dsRNA during viral infections (76). Furthermore, HDAC6-dependent RIG-I deacetylation also regulates RIG-I oligomerization upon ligand binding, thus facilitating RIG-I activation (75).

#### Other Regulatory Mechanisms

RIG-I signal transduction is further regulated by additional PTMs, regulatory proteins, and other cellular processes (Figure 2). It is worth noting that a number of UBL proteins including SUMO, ISG15, FAT10, and Atg8–Atg12 are involved in these positive or negative regulatory mechanisms (77). SUMOylation serves as a positive regulator of RIG-I by enhancing the RIG-I and MAVS binding (78). On the contrary, the HLA-F adjacent transcription 10 (FAT10), an UBL modifier protein, was shown to negatively regulate RIG-I by modulating RIG-I solubility through a non-covalent association with CARDs (79). In addition, IFN-induced ISG15 negatively regulates the RIG-I mediated signaling in a feedback-loop control manner (80). SEC14L1 has been observed competing with MAVS for RIG-I CARD binding (81). Furthermore, autophagy has been reported to be involved in RIG-I modulation through its key regulator, the Atg5–Atg12 conjugate. Atg5–Atg12 has been found to suppress RIG-I–MAVS interaction, thereby inhibiting downstream signaling (82). Recently, deamidation of CTD has been described as a distinct means to induce RIG-I activation. For examples, vGAT (glutamine amidotransferase), from KSHV (kaposi’s sarcoma-associated herpesvirus) and γHV68 (murine gamma herpesvirus 68), recruits cellular phosphoribosylformyglycinamide synthase to deamindate and activate RIG-I (83, 84).

## Viral Antagonism of RIG-I Signaling

In order to establish infections, viruses have developed sophisticated mechanisms to counteract host immune responses. With regard to RIG-I signaling, these include mechanisms such as altering viral genomes and their intermediate transcripts to avoid detection, manipulating the activation and degradation of RIG-I and MAVS, as well as modulating downstream signaling cascades. Studying these antagonistic viral strategies has greatly broadened our understanding of RIG-I activation and regulation.

### Sequestration of Viral RNAs

Since 5′ triphosphate (5′ppp) is an important feature recognized by RIG-I, modification of this motif has long been described as one of the major mechanisms for viruses to antagonize RIG-I signaling. Crimean–Congo hemorrhagic fever virus, Borna disease virus (BDV), and hantavirus (HTNV) remove the 5′ppp group on their genome posttranscriptionally, make RIG-I unable to bind to viral RNA, and therefore incapable of triggering RIG-I activation (85). Mechanistically, HTNV uses the “prime and realign” strategy to generate a 5′-terminal monophosphorylate (86, 87). BDV on the other hand, employs genome trimming to form a 3′-terminal overhang as well as convert 5′ppp to 5′p to avoid detection by RIG-I (88). The arenavirus presents an unpaired 5′ppp-nucleotide overhang to evade recognition by RIG-I (89). The 5′-end of viral RNA can also be modified through RNA-capping pathways. For example, the genomic RNA of polioviruses linked to Vpg (viral protein genome-linked) to cap the 5′-end from exposure to RIG-I (90). The 5′-end capping with 7-methyl guanosine and methylation of 5′ppp dsRNA at the 2′-*O* position makes viral RNA non-distinguishable from the host mRNAs, and therefore does not stimulate RIG-I (91, 92).

By contrast, some viruses encode viral proteins to prevent RNA recognition. The EBOV utilizes its VP35 protein to sequester viral RNA (18). The crystal structural analysis indicates that the VP35 interferon inhibitory domain competes with RIG-I for dsRNA binding by forming an “end-cap” complex with dsRNA, resulting in substantially diminished activation of RIG-I (93). Similarly, the marburg virus VP35 spirals around the dsRNA backbone and end-caps the dsRNA to escape from RIG-I detection (94, 95). The IAV non-structural protein 1 (NS1) possesses dsRNA-binding properties to shield viral RNA from RIG-I (96). IAV has also been shown to antagonize RIG-I activation *via* its viral polymerase subunit PB2. PB2 position 627K in the mammalian strain increases PB2-nucleocapids binding affinity, thus inhibiting RIG-I interaction with the nucleoprotein-encapsidated 5′ppp RNA (22, 97).

In addition to altering and concealing their genome to prevent RNA binding, viruses also re-localize viral RNA to specific cellular compartments, such as mitochondria, endoplasmic reticulum (ER), and Golgi, to avoid cytosolic surveillance by RIG-I. For instance, the DENV conceals dsRNA in the intracellular membrane as an escape strategy (98). ER is an important organelle for viral entry, replication, and assembly. The severe acute respiratory syndrome (SARS) coronavirus (SARS-CoV) has been shown to induce a modified ER to hide its replicating RNA from detection (99). These viral antagonism strategies highlight the importance of cellular organelle localization in viral–host interactions during innate antiviral responses.

### Manipulation of RIG-I–MAVS Signaling

#### Modulation of the PTMs

**Figure 3:**
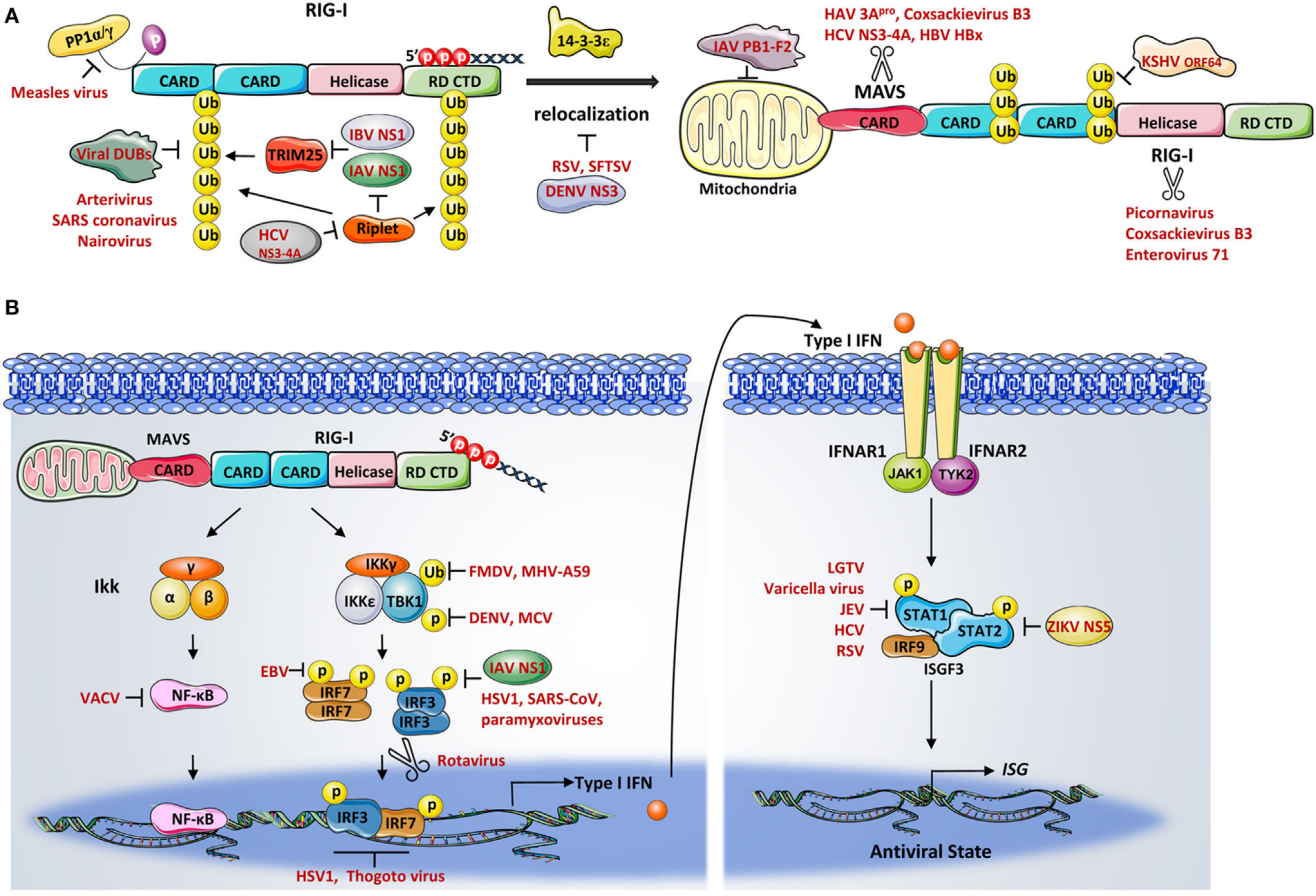
**(A)** Viral inhibition of retinoic acid-inducible gene-I (RIG-I)– mitochondrial antiviral signaling protein (MAVS) signaling. Numerous viral proteins have been described as RIG-I signaling antagonists which inhibit the activation of RIG-I and its adaptor protein MAVS. Influenza A virus (IAV) NS1 and influenza B virus NS1 were both shown to suppress the TRIM25-mediated ubiquitination of RIG-I caspase activation and recruitment domains. The protease NS3-4A of hepatitis C virus functions by targeting Riplet. A number of virus-encoded deubiquitination enzymes from various viruses remove Lys63-linked ubiquitin from RIG-I, resulting in signaling suppression. The measles virus efficiently blocks RIG-I dephosphorylation to prevent RIG-I activation. Dengue virus (DENV), respiratory syncytial virus, and thrombocytopenia syndrome virus (SFTSV) were involved in disrupting RIG-I translocation to the mitochondria. Numerous virus-encoded proteases were shown to directly cleave or degrade RIG-I and MAVS. Additionally, the PB1-F2 of IAV decreases the mitochondrial membrane potential thereby inhibiting RIG-I–MAVS interaction. **(B)** Viral modulation of downstream signaling components. Ubiquitination- and phosphorylation-mediated TBK1 activation were shown be antagonized by a number of viruses, including foot-and-mouth disease virus, mouse hepatitis virus A59, molluscum contagiosum virus, and DENV. IAV NS1, herpes simplex virus type 1, severe acute respiratory syndrome coronavirus (SARS-CoV), and several paramyxoviruses have been shown to interfere with interferon regulator factor (IRF) 3 phosphorylation. Epstein–Barr virus suppresses IRF7 transcriptional activity. The rotavirus targets both IRF3 and IRF7 for degradation. The binding of transcription factors to the IFNB promoter is also challenged by herpes simplex virus and the thogoto virus. Other viruses subvert the JAK–STAT signaling to inhibit the production of interferon-stimulated genes. STAT1 and STAT2 are therefore often targets by a number of viruses.

As reviewed above, ubiquitination represents one critical PTM mechanism of RIG-I activation and, not surprisingly, is an attractive target for viral manipulation (Figure 3A). Viruses have evolved ways to inhibit K63-linked ubiquitination of RIG-I by interacting with the E3 ligases TRIM25 and Riplet. For instance, IAV NS1 from various strains has been shown to suppress TRIM25-mediated RIG-I CARDs ubiquitination. Among all the TRIM25 binding amino acids identified in NS1, R38/K41 and E96/E97 were described as critical in interfering with the coil-coiled domain of TRIM25. These interactions resulted in an inhibition of TRIM25 multimerization and therefore blocked the RIG-I CARDs ubiquitination (100). Intriguingly, NS1-TRIM25 binding is found to be preserved in human and avian, but lost in mouse, indicating a species-specific manner of inhibition. This study further demonstrates that the NS1 suppression of RIG-I ubiquitination in mouse is Riplet-dependent (101). Conversely, phosphorylation of NS1 at Thr49 was recently identified as impairing the NS1–TRIM25 interaction, thereby suppressing its antagonistic activity of RIG-I signaling (102). Phosphorylation of another site on NS1, Thr80, has also been reported to disrupt NS1 binding affinity with RIG-I (103). Similar to IAV, the IBV non-structural NS protein (NS1-B) has recently been described as inhibiting RIG-I ubiquitination, which involves TRIM25-NS1 C-terminal effector domain interaction and the RIG-I/TRIM25/NS1-B complex formation (104). By contrast, the protease NS3-4A of HCV functions differently, rather than inhibiting TRIM25, it is thought to target the E3 ligase Riplet. NS3-4A directly disrupts Riplet, abolishes Riplet-mediated RIG-I ubiquitination, and further reduces the interaction between TRIM25 and RIG-I (54).

On the other hand, some viruses encode enzymes that directly deubiquitinate RIG-I. For instance, KSHV encoded deubiquitinase ORF64 cleaves Lys63-ubiquination chains on CARDs, blocks CARDs interaction between RIG-I and MAVS, thereby downregulating RIG-I signaling (105). Other viruses including arterivirus, nairovirus, SARS-CoV, and foot-and-mouth disease virus (FMDV) have also been reported to downregulate RIG-I ubiquitination through their viral encoded DUBs (106, 107).

Few viruses have been shown to manipulate RIG-I regulation with regards to targeting the phosphorylation or dephosphorylation process of RIG-I. Nevertheless, it was reported that MV efficiently escapes antiviral response *via* suppressing RIG-I dephosphorylation in dendritic cells (DCs). In this study, the growth arrest and DNA damage protein (GADD34) was shown to form complexes with PP1 to facilitate RIG-I activation. The MV infection induced DC-SIGN signaling results in an inhibition of GADD34-PP1 phosphatases activity and thereby impairs RIG-I activation (108).

### Degradation of RIG-I and MAVS

Another distinct strategy used by viruses to antagonize RIG-I signaling is the direct cleavage or degradation of the receptor and multiple members of the signaling cascade (Figure 3A). RIG-I has been reported in some studies to be cleaved by the proteinase 3C^pro^ during infections with picornavirus, coxsackievirus B3 (CVB3), and enterovirus 71 (EV71) (109, 110). The encephalomyocarditis virus directs both caspase- and proteasome-dependent degradation of RIG-I (111). Intriguingly, the NS1–NS2 degradasome of the respiratory syncytial virus (RSV) has been shown to mediate the proteasomal degradation of RIG-I (112).

Mitochondrial antiviral signaling protein is also a well-studied molecule which is often targeted by many types of viral-induced cleavage. For example, the hepatitis A virus (HAV) cleaves MAVS for proteolysis by its protease 3C^pro^ (113). Both CVB3 proteinase 2A^pro^ and 3C^pro^ trigger MAVS cleavage at different sites during infection, and the cleavage of MAVS by EV71 is accomplished *via* its 2A^pro^ activity (110, 114). In addition, serine protease NS3-4A of HCV cleaves MAVS, removing it from the mitochondria, thereby inhibiting downstream signaling (36, 115). In a parallel fashion, many viruses mediate cellular proteolytic degradation of MAVS to attenuate RIG-I antiviral responses. Hepatitis B virus viral protein HBx triggers the proteasome-mediated degradation of MAVS through Lys136 ubiquitination (116). Another study reported that the HAV cysteine protease ABC targets MAVS for proteolysis at mitochondrial membrane (113). Additionally, viral modulation of cellular organelles such as mitochondria also affects RIG-I–MAVS signaling. The PB1-F2 of IAV, for instance, has been described as decreasing the mitochondrial membrane potential, resulting in the acceleration of mitochondrial fragmentation, thereby inhibiting RIG-I–MAVS signaling (117–119).

It is important to note that the proper localization of RIG-I and MAVS is a prerequisite for effective signaling transduction. MAVS resides on the mitochondrial membrane, peroxisomes, and mitochondria-associated membranes for antiviral signaling. In fact, a RIG-I translocon has been identified to direct RIG-I redistribution from cytosol to membranes during viral infection (49). Studies have shown that several viruses encode proteins to disrupt the proper localization of RIG-I or MAVS as a novel mechanism of regulation, such as NS3 of DENV (113), nucleoprotein of RSV (120), and non-structural proteins of thrombocytopenia syndrome virus (SFTSV) (121).

### Modulation of Downstream Signaling Components

To ensure successful RIG-I signaling transduction, the kinase activities of TBK1 and IKKϵ are tightly controlled *via* various regulatory mechanisms and are common targets of viruses (Figure 3B). For example, both the leader proteinase (L^pro^) of FMDV (122) and the non-structural protein 3 (ns3) of the mouse hepatitis virus A59 (123) inhibit ubiquitination of TBK1. Dengue virus serotype4 non-structural proteins, NS2A and NS4B, as well as the FLIPs proteins encoded by the molluscum contagiosum virus (MCV), all reduce TBK1 phosphorylation, thereby preventing its activation (124, 125). Several viruses have been shown to prevent the formation of functional TBK1-containing complexes. The K7 protein of the VACV prevents TBK1/IKKϵ complex-induced IRF activation by targeting host DEAD box protein 3 (DDX3) (126). Two other viruses, the NY-1 HTNV and SARS-CoV, disrupt the TBK1–TRAF3 and TANK–TBK1/IKKϵ complex, respectively (127, 128). Moreover, SFTSV has been shown to irreversibly re-localize TBK1 and IKK from mitochondria and sequester the TBK1/IKKϵ/IRF3 complex *via* the formation of inclusion bodies, causing signaling cascade termination (129).

Viral regulation of the transcription factors, IRFs and NF-κB, further serve as points of control in RIG-I signaling (Figure 3B). One of the best studied examples is the inhibition of IRF3 activity by the IAV NS1 protein (130). Besides this, the HSV-1, rabies virus, SARS-CoV, as well as several paramyxoviruses have been demonstrated to interfere with the phosphorylation state of IRF3, thereby blocking IFN induction (131–134). The EBV conjugates SUMO to IRF7 at lysine 452 to decrease IRF7 transcriptional activity (135). The rotavirus NS1, targets both IRF3 and IRF7 for degradation to prevent IRFs from undergoing dimerization (136). Viruses have also developed various means to suppress the IRF3 DNA binding ability. Herpes simplex virus, thogoto virus, and KSHV, all developed strategies to downregulate IRF3 transcriptional activity by either disrupting IRF3 binding complex formations or competing binding regions on the IFNB promoter (137–139). Viral strategies in inhibiting cytoplasmic or transcriptional activities of NF-κB have been extensively studied during the VACV infection. Studies reported that multiple proteins encoded by VACV and HSV-1 suppress NF-κB activation (140–143).

Viruses have also developed multiple inhibitory mechanisms to counteract the IFN stimulation of ISGs by targeting STAT1 and/or STAT2 (Figure 3B). For example, the langat virus was shown to inhibit the phosphorylation of both STAT1 and STAT2 (144). Varicella viruses and the Japanese encephalitis virus, both block the JAK/STAT1 pathway through multiple mechanisms including inhibiting STAT proteins phosphorylation and nucleotranslocation (145, 146). The non-structural protein NS5 of several flaviviruses, have been shown to target STAT proteins *via* distinct mechanisms. For example, MNV NS5 inhibits STAT1 phosphorylation, whereas DENV NS5 interacts with UBR4 to promote STAT2 degradation (147, 148). By contrast, the Zika virus NS5 induced proteasomal degradation of STAT2 was recently identified as UBR4 independent (149). Furthermore, other viruses, such as HCV (150), RSV (151), and paramyxovirus (152), also demonstrate negative regulation of the JAK–STAT pathway.

## Concluding Remarks

Studies from the past decade have well established RIG-I as one of the principal PRRs for the recognition of cytoplasmic viral RNA, as well as defining its critical role in the induction of IFNs during viral infections. Our understanding of the RIG-I-mediated antiviral response has been greatly expanded with the key discoveries made regarding the molecular mechanism of RIG-I regulation, such as ubiquitination, phosphorylation, and acetylation. Meanwhile, investigating viral strategies to manipulate RIG-I responses not only allow us to understand the viral pathogenesis, but also significantly contributed to our knowledge of how RIG-I is activated and regulated. These new insights into the viral-mediated RIG-I regulations are important for vaccine and drug development aiming to suppress infectious diseases and enhance immune responses.

## Author Contributions

YL wrote the manuscript. RL and DO revised and approved the manuscript.

## Conflict of Interest Statement

The authors declare that the research was conducted in the absence of any commercial or financial relationships that could be construed as a potential conflict of interest.
